# *Ziphius cavirostris* presence relative to the vertical and temporal variability of oceanographic conditions in the Southern California Bight

**DOI:** 10.1002/ece3.11708

**Published:** 2024-07-14

**Authors:** Clara M. Schoenbeck, Alba Solsona‐Berga, Peter J. S. Franks, Kaitlin E. Frasier, Jennifer S. Trickey, Catalina Aguilar, Isaac D. Schroeder, Ana Širović, Steven J. Bograd, Ganesh Gopalakrishnan, Simone Baumann‐Pickering

**Affiliations:** ^1^ Scripps Institution of Oceanography University of California San Diego, La Jolla California USA; ^2^ National Oceanic and Atmospheric Administration Pacific Islands Fisheries Science Center Honolulu Hawaii USA; ^3^ Institute of Marine Sciences University of California Santa Cruz California USA; ^4^ Department of Biology Norwegian University of Science and Technology Trondheim Norway; ^5^ Environmental Research Division, Southwest Fisheries Science Center, National Marine Fisheries Service National Oceanic and Atmospheric Administration Monterey California USA

**Keywords:** Cuvier's beaked whales, echolocation clicks, El Niño, habitat model, optimum multiparameter analysis, passive acoustic monitoring, Southern California Bight, water masses

## Abstract

The oceanographic conditions of the Southern California Bight (SCB) dictate the distribution and abundance of prey resources and therefore the presence of mobile predators, such as goose‐beaked whales (*Ziphius cavirostris*). Goose‐beaked whales are deep‐diving odontocetes that spend a majority of their time foraging at depth. Due to their cryptic behavior, little is known about how they respond to seasonal and interannual changes in their environment. This study utilizes passive acoustic data recorded from two sites within the SCB to explore the oceanographic conditions that goose‐beaked whales appear to favor. Utilizing optimum multiparameter analysis, modeled temperature and salinity data are used to identify and quantify these source waters: Pacific Subarctic Upper Water (PSUW), Pacific Equatorial Water (PEW), and Eastern North Pacific Central Water (ENPCW). The interannual and seasonal variability in goose‐beaked whale presence was related to the variability in El Niño Southern Oscillation events and the fraction and vertical distribution of the three source waters. Goose‐beaked whale acoustic presence was highest during the winter and spring and decreased during the late summer and early fall. These seasonal increases occurred at times of increased fractions of PEW in the California Undercurrent and decreased fractions of ENPCW in surface waters. Interannual increases in goose‐beaked whale presence occurred during El Niño events. These results establish a baseline understanding of the oceanographic characteristics that correlate with goose‐beaked whale presence in the SCB. Furthering our knowledge of this elusive species is key to understanding how anthropogenic activities impact goose‐beaked whales.

## Graphical abstract

Goose‐beaked whales are deep‐diving marine mammals, whose elusive behavior presents challenges for studying them. This research utilizes passive acoustics to investigate the seasonal and interannual trends in their presence within the Southern California Bight. By modeling acoustic presence with water masses and El Niño Southern Oscillation events, the study reveals increased whale presence in nutrient‐rich winter and spring compared to nutrient‐poor late summer and fall, as well as elevated presence during El Niño.

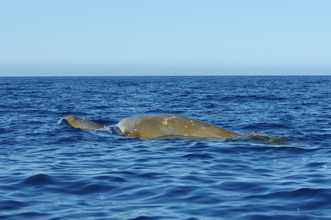

## 1 INTRODUCTION

**FIGURE 1:**
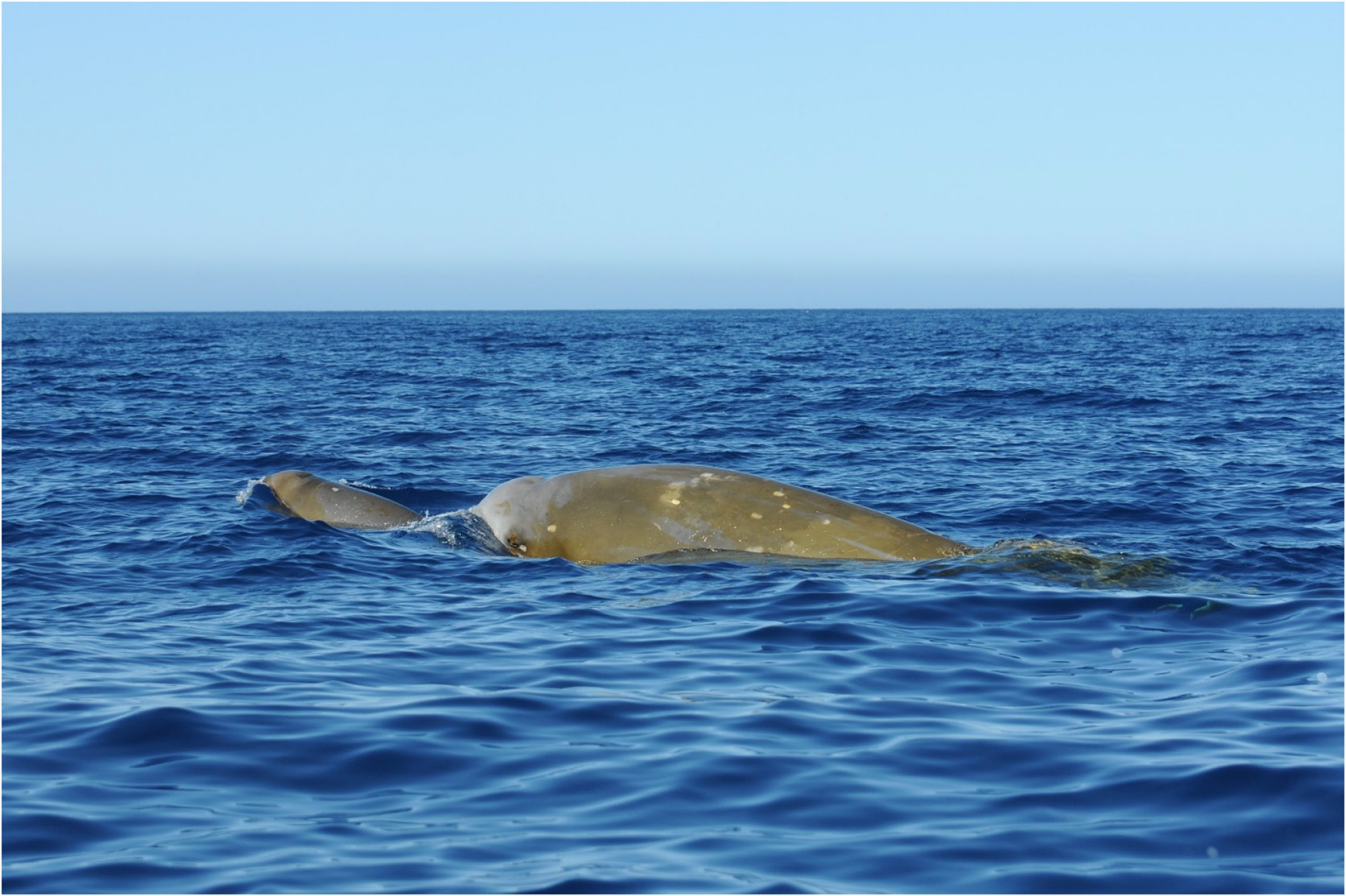
Goose‐beaked whale mom and calf. Image taken by Jennifer S. Trickey in the Guadalupe Island Biosphere Reserve under SEMARNAT permits SGPA/DVGS/09096/16 and SGPA/DVGS/02740/17.

Goose‐beaked whales (*Ziphius cavirostris*, Figure 1) are widely distributed and found in tropical to subarctic waters (Heyning & Mead, 2009). However, with most of their time spent at depth and a low profile while at the surface, visual surveys are inefficient for gathering abundance and distribution data (Barlow, 2015). Goose‐beaked whales, like other odontocetes, produce echolocation signals while foraging (Johnson et al., 2004). These high‐frequency signals allow them to detect their prey (Au, 1993). Beaked whales produce frequency‐modulated (FM) upsweep pulses that can be identified at a species level by their spectral and temporal characteristics, such as signal duration, mean spectra, waveform, inter‐pulse interval (IPI), and peak frequency (Baumann‐Pickering et al., 2013). Their acoustic activity during foraging dives and our capability to identify species by their echolocation FM pulses make beaked whales prime candidates for passive acoustic monitoring (PAM). PAM offers a non‐invasive method for gathering long‐term acoustic data at remote sites (Wiggins & Hildebrand, 2007). Long‐term monitoring is key to informing conclusions about beaked whale seasonal patterns in presence, geographical distributions, and abundance trends.

Stomach content analysis has revealed that goose‐beaked whales feed on cephalopods, fish, and crustaceans; however, their diets are primarily composed of cephalopods (West et al., 2017). Within the Southern California Bight (SCB) region, prey patches are heterogeneously distributed (Benoit‐Bird et al., 2020; Southall et al., 2019). With the highly energetic cost of performing such deep dives, prey hotspots are essential for goose‐beaked whale fitness and survival (Benoit‐Bird et al., 2020; Southall et al., 2019). Given the limited information on goose‐beaked whale prey, this paper investigates the physical oceanographic factors that best describe variability in goose‐beaked whale acoustic presence in the SCB and potential habitat preferences, which might also shape their prey community.

Goose‐beaked whales found in the SCB are frequently present around the canyon slopes and basins near San Clemente Island year‐round (Schorr et al., 2014). Tagged whales and repeated sightings of photo‐identified individuals during visual efforts suggests that goose‐beaked whales demonstrate some site fidelity in the region (Schorr et al., 2014). This region also features the United States Navy's Southern California Anti‐Submarine Warfare Range (SOAR), utilized by the US military for regular training exercises (Falcone et al., 2009, 2017). Goose‐beaked whales are sensitive to anthropogenic sound, with sublethal consequences in response to mid‐frequency active sonar (MFAS) (Cox et al., 2006; D'Amico et al., 2009; Falcone et al., 2017; Filadelfo et al., 2009). Tagging studies in the region demonstrated that goose‐beaked whales inhabiting the SOAR changed their diving behaviors in response to the use of MFAS (Falcone et al., 2017). The combined interest in the region as a prime goose‐beaked whale foraging ground and navy training site underscores the importance of understanding the spatio‐temporal patterns in goose‐beaked whale presence, especially with increasing anthropogenic sound impacts within the SCB.

The SCB is a highly productive region, defined by its complex bathymetry formed by the various islands and canyons scattered within the SCB, and the presence of multiple source waters (Hickey, 1993). Within the SCB, three main water masses can be defined: the Pacific Subarctic Upper Water (PSUW), the Eastern North Pacific Central Water (ENPCW), and the Pacific Equatorial Water (PEW) (Hickey, 1993). PSUW is characterized by low temperature, low salinity, high dissolved oxygen, and high nutrients (Lynn & Simpson, 1987). The PSUW also reflects the equatorward flowing California Current within the California Current System (Bograd et al., 2019). ENPCW is a surface water mass characterized by high temperatures, high salinity, low dissolved oxygen, and low nutrients (Lynn & Simpson, 1987). The ENPCW enters the California Current System from the west, bringing in less productive offshore water into the SCB (Hickey, 1993). PEW is a subsurface water mass, characterized by high temperature, high salinity, low dissolved oxygen, and high nutrients (Lynn & Simpson, 1987). The PEW enters the California Current System from the south and reflects the poleward flowing California Undercurrent (Hickey, 1993; Lynn & Simpson, 1987).

Within the SCB, the southward‐flowing California Current and northward‐flowing California Undercurrent are prevalent throughout the year (Bograd et al., 2019). However, the strength and distribution of the currents within the SCB varies seasonally and interannually (Bograd et al., 2019). Across most of the SCB, the California Current (transporting PSUW) has a seasonal southward flow maximum during the summer (Hickey, 1979) and the California Undercurrent (transporting PEW) reaches its seasonal northward flow maximum in late summer and an additional flow maximum in winter (Hickey, 1993). Within the southern portion of the SCB, the California Undercurrent has a seasonal flow maximum during the late summer and fall and weakens during the spring (Hickey, 1979). During the winter and spring seasons, the southwest corner of the SCB has a strong seasonal influx of offshore North Pacific gyre water (defined as the ENPCW) (Bograd et al., 2019). The seasonal injection of the ENPCW is strongest in the offshore waters but appears within the SCB as it is entrained by the poleward‐flowing California Undercurrent (Bograd et al., 2019). There are also interannual patterns in current strength and water mass distribution in relation to El Niño Southern Oscillation (ENSO) events. During El Niño, there is a weakening of equatorward winds that results in weaker upwelling of nutrient‐rich waters into the euphotic zone (Checkley & Barth, 2009; Jacox et al., 2015). La Niña events bring opposite trends, characterized by anomalously colder water and strengthened upwelling (Jacox et al., 2015). These long‐term patterns dictate the physical oceanography and therefore productivity of the SCB; their impacts are felt up the food web and may modulate the presence of top predators such as goose‐beaked whales.

To investigate the oceanographic variables that best describe changes in goose‐beaked whale presence, this study combines oceanographic data from a data‐assimilating state‐estimate model, with long‐term passive acoustic recordings. In this paper, we investigate goose‐beaked whale presence in relation to the physical oceanography of the SCB. The findings are key to understanding the ecology of these elusive deep divers, especially in light of increasing anthropogenic noise.

## 2 MATERIALS AND METHODS

### 2.1 Acoustic recordings

**FIGURE 2:**
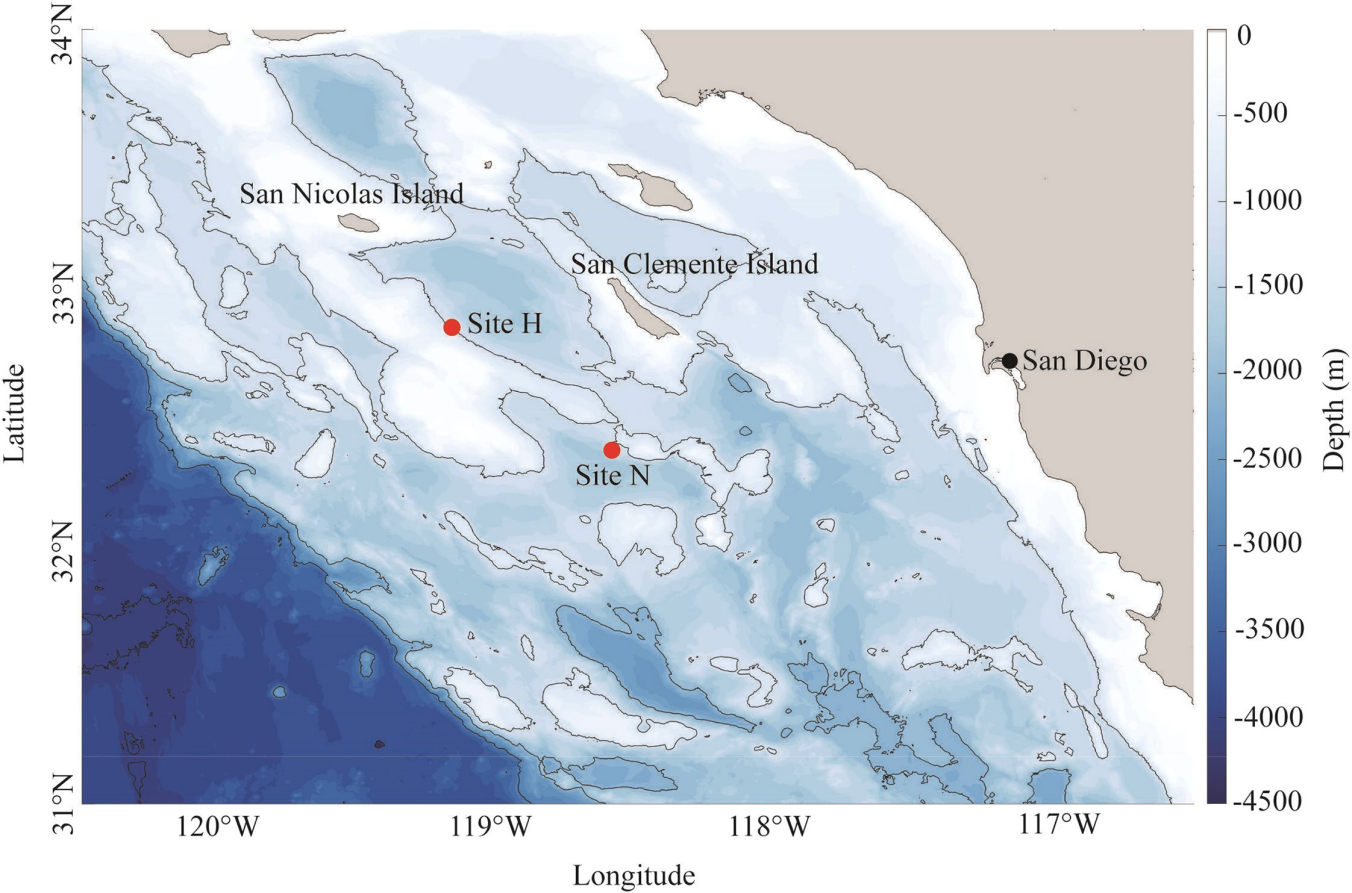
Map of the study sites in the SCB. Sites H and N are indicated by red dots. Land is indicated in gray. Depth is represented by the blue color bar, with contour lines at every 1000 m. Bathymetry data are from Ryan et al. (2009).

Acoustic data were collected using High‐frequency Acoustic Recording Packages (HARPs, Wiggins & Hildebrand, 2007) deployed in the SCB at site H, southeast of San Nicolas Island, and site N, south of San Clemente Island (Figure 2, Table S1). HARPs were comprised of a hydrophone suspended 10–30 m above the seafloor, a data logger, battery packs, and acoustic releases for recovery. Anchored to the seafloor, HARPs passively recorded the ocean soundscape with a 200 kHz sampling frequency and 16‐bit quantization, resulting in an effective bandwidth of 10 Hz to 100 kHz. Each HARP hydrophone was calibrated in the laboratory before its initial deployment, while representative full systems were also calibrated at the US Navy's Transducer Evaluation Center facility to verify the laboratory calibrations. Consistent deployments provided a near‐continuous time series at both acoustic monitoring sites. Acoustic data was collected from July 2007 through September 2020 at site H, and from January 2009 through September 2020 at site N. Any gaps in the time series were due to battery life, data storage capacity, system failure, and/or vessel and crew availability to service the instruments.

### 2.2 Data processing

Goose‐beaked whale echolocation signals were identified using a combination of automated detection and manual verification techniques (Baumann‐Pickering et al., 2014). All echolocation clicks were first identified using the automated Teager Kaiser energy detector (Roch et al., 2011; Soldevilla et al., 2008). Individual click detections were then filtered with a 10‐pole Butterworth band‐pass filter with a passband between 5 and 95 kHz. The acoustic presence of beaked whale FM echolocation pulses was determined by investigating 75 s segments for a minimum requirement of seven detections within the segment. These segments were then classified as containing beaked whale presence if more than 13% of all initially detected signals had peak and center frequencies above 32 and 25 kHz, a duration longer than 355 μs, and an upsweep rate of more than 23 kHz/ms (Baumann‐Pickering et al., 2013).

The automatically detected beaked whale clicks were then verified using *DetEdit*, an open‐source software for visualizing events within large acoustic datasets (Solsona‐Berga et al., 2020). Once goose‐beaked whale clicks were verified by a trained analyst, the time of each click was used to designate click positive minutes. The number of click positive minutes was then summed for each month. Goose‐beaked whale presence data was determined as the number of click positive minutes recorded. Total monthly minutes of goose‐beaked whale presence were used to examine the seasonal and interannual patterns in presence. Finally, to account for variations in recording efforts over time at each site, minutes of presence were divided by percent effort for each month.

### 2.3 Environmental data

Daily oceanographic data was obtained from the California State Estimate ‐ Short‐Term State Estimate (CASE‐STSE) product over January 2007 to September 2020 (http://www.ecco.ucsd.edu/case.html). The CASE‐STSE utilizes a regional implementation of the Massachusetts Institute of Technology‐ general circulation model (MITgcm; Marshall, Adcroft, et al., 1997; Marshall, Hill, et al., 1997) and the Estimating the Circulation and Climate of the Ocean (ECCO; Stammer et al., 2002) four‐dimensional variational (4D‐Var) assimilation system to assimilate a variety of observations including satellite along‐track sea surface height (SSH) and gridded sea surface temperature (SST), temperature and salinity profiles from Spray gliders and Argo profilers, temperature profiles from repeat high‐resolution expendable bathythermograph (XBT), temperature profiles from autonomous pinniped bathythermograph (APB) and shipboard conductivity, temperature, and depth (CTD) profiles. CASE‐STSE is produced by merging a series of nonoverlapping 3‐month state estimates over the study period. Each 3‐month state estimate uses the Hybrid Coordinate Ocean Model (HYCOM; Chassignet et al., 2007) 1/12° global daily analysis as the first‐guess (“prior”) model solution, and minimizes the model‐data misfit to produce the optimized state by adjusting the model controls via iterative optimization. CASE‐STSE product is validated and used to study annual and interannual variability and volume and heat budgets in the California Current System (CCS; Zaba et al., 2018, 2020). A detailed description of the model and validation of CASE‐STSE is provided in Zaba et al. (2018). The CASE‐STSE model domain extends from 27° N to 40° N and from 130° W to 114° W, with a horizontal resolution of 1/16 degrees (~8 km) and 72 vertical depth levels with level spacing gradually increasing from 5 m near the surface to 200 m near the bottom at 5450 m. However, CASE‐STSE solutions below 500 m were not included in the present work due to the limited availability of observational data below 500 m. CASE‐STSE provides daily averaged SSH, and three‐dimensional fields for temperature, salinity, and horizontal and vertical velocities over the study period. Daily and monthly values of CASE‐STSE solutions were averaged across a 5 × 5 km square around each HARP site at 45 m to capture the variability in the local near‐surface oceanography.

In this work, El Niño Southern Oscillation (ENSO) events were investigated, as they are a significant contributor to interannual environmental variability. La Niña and El Niño events were quantified using the Oceanic Niño Index (ONI), a three‐month running average of the ERSST.v5 SST anomalies calculated from the Niño 3.4 region. This index was downloaded from the NOAA National Weather Service Climate Prediction Center (https://origin.cpc.ncep.noaa.gov/products/analysis_monitoring/ensostuff/ONI_v5.php).

### 2.4 Optimum multiparameter analysis

Optimum multiparameter (OMP) analysis utilizes hydrographic data to quantify the fraction of predefined source waters over time and space from local water properties (Tomczak & Large, 1989). OMP analysis assumes temperature and salinity are conservative when mixed (Liu & Tanhua, 2021; Tomczak & Large, 1989). Before performing the OMP analysis, the desired source waters must be defined (Bograd et al., 2019). Following Bograd et al. (2019), ENPCW, PSUW, and PEW were defined by the temperature and salinity values (denoted by *T*_PEW/PSUW/ENPCW_ and *S*_PEW/PSUW/ENPCW_ in Equations 1 and 2) at the depths with the strongest contribution of each source water (Table S2). Given these source water definitions (*T*_PEW/PSUW/ENPCW_ and *S*_PEW/PSUW/ENPCW_), the OMP analysis used three systems of linear equations to characterize modeled temperature (*T*_MODEL_) and salinity (*S*_MODEL_) data, solving for the relative source water contributions (fractions of source waters, denoted by (*X*_PEW/PSUW/ENPCW_). *T*_MODEL_ and *S*_MODEL_ were taken from the CASE‐STSE product at gradually increasing incremental depth intervals from 5 to 500 m and daily intervals. Vertical and temporal replicates are denoted by *d*, depth, and *t*, time:

(1) $$X_{\mathrm{PEW}\left(d,t\right)}T_{\mathrm{PEW}}+X_{\text{PSUW}\left(d,t\right)}T_{\text{PSUW}}+X_{\text{ENPCW}\left(d,t\right)}T_{\text{ENPCW}}=T_{\text{MODEL}\left(d,t\right)}+R_{T\left(d,t\right)}$$

(2) $$X_{\mathrm{PEW}\left(d,t\right)}S_{\mathrm{PEW}}+X_{\text{PSUW}\left(d,t\right)}S_{\text{PSUW}}+X_{\text{ENPCW}\left(d,t\right)}S_{\text{ENPCW}}=S_{\text{MODEL}\left(d,t\right)}+R_{S\left(d,t\right)}$$

(3) $$X_{\mathrm{PEW}\left(d,t\right)}+X_{\text{PSUW}\left(d,t\right)}+X_{\text{ENPCW}\left(d,t\right)}=1+R_{\sum \left(d,t\right)}$$

**FIGURE 3:**
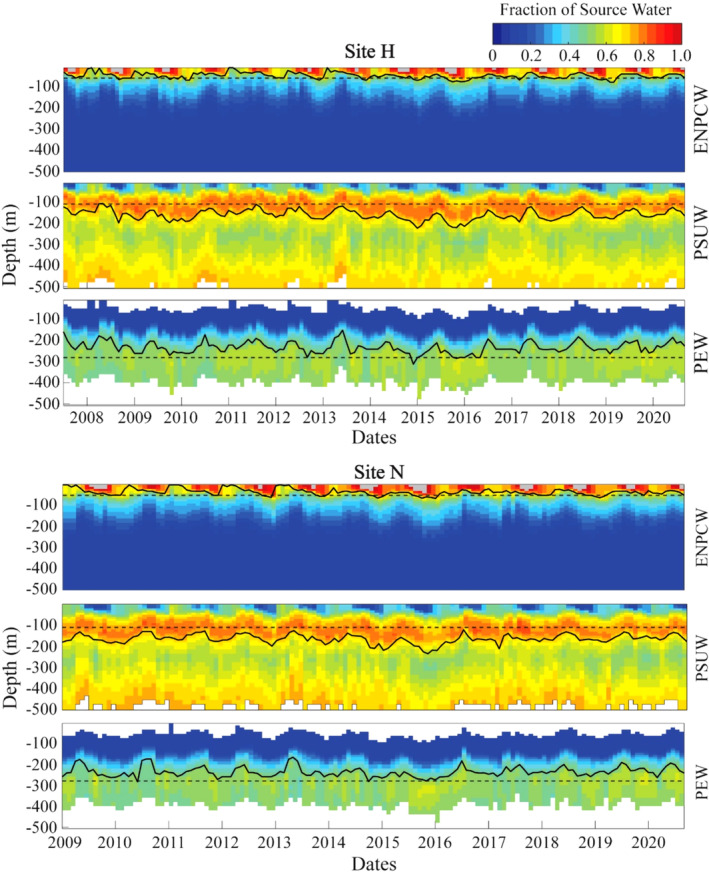
Fractions of source waters and vertical distributions over time. Top: Site H. Bottom: Site N. A time series of each source water with depth on the *y*‐axis and year on the *x*‐axis. Plots are colored by the fraction of each water mass present at that time and depth. The dashed black line marks the selected depth (depth of the maximum average fraction) at which the fraction of each source water was used to quantify the source waters. The solid black line traces the selected fraction (upper quartile fraction of the averaged fractions at each depth) and quantifies the change in depth of each water mass. Times when the fraction of ENPCW exceeded one are in gray.

Relative source water contributions (*X*_PEW/PSUW/ENPCW_) were calculated using a non‐negative least squares method with the aims to minimize residuals (*R*) (Bograd et al., 2019; Liu & Tanhua, 2021; Tomczak & Large, 1989). The equations were also normalized and weighted following the method outlined in Tomczak and Large (1989), with the mass conservation equation (3) assigned the same weight as the temperature equation. These calculations were performed using modified code from the MATLAB OMP toolbox (https://www.mathworks.com/matlabcentral/fileexchange/1334‐omp‐analysis)(Karstensen, 2024). OMP analysis was run for both sites H and N, and yielded a fraction of each source water for day and depth from 0 to 500 m. The error matrix of the mass residuals included discrepancies in the resolution of the environmental data and general environmental variability (Tomczak & Large, 1989). Times and depths when the residuals exceeded 2.5% were removed from the dataset (Hinrichsen & Tomczak, 1993). In a few cases, the totaled fractions of ENPCW near the surface were greater than one due to remaining uncertainty in the analysis. The ENPCW fractions in these cases were within one standard deviation of one (1 + 0.2355 at site H, 1 + 0.2646 at site N) and are shown in gray in the plotted timeseries (Figure 3).

**FIGURE 4:**
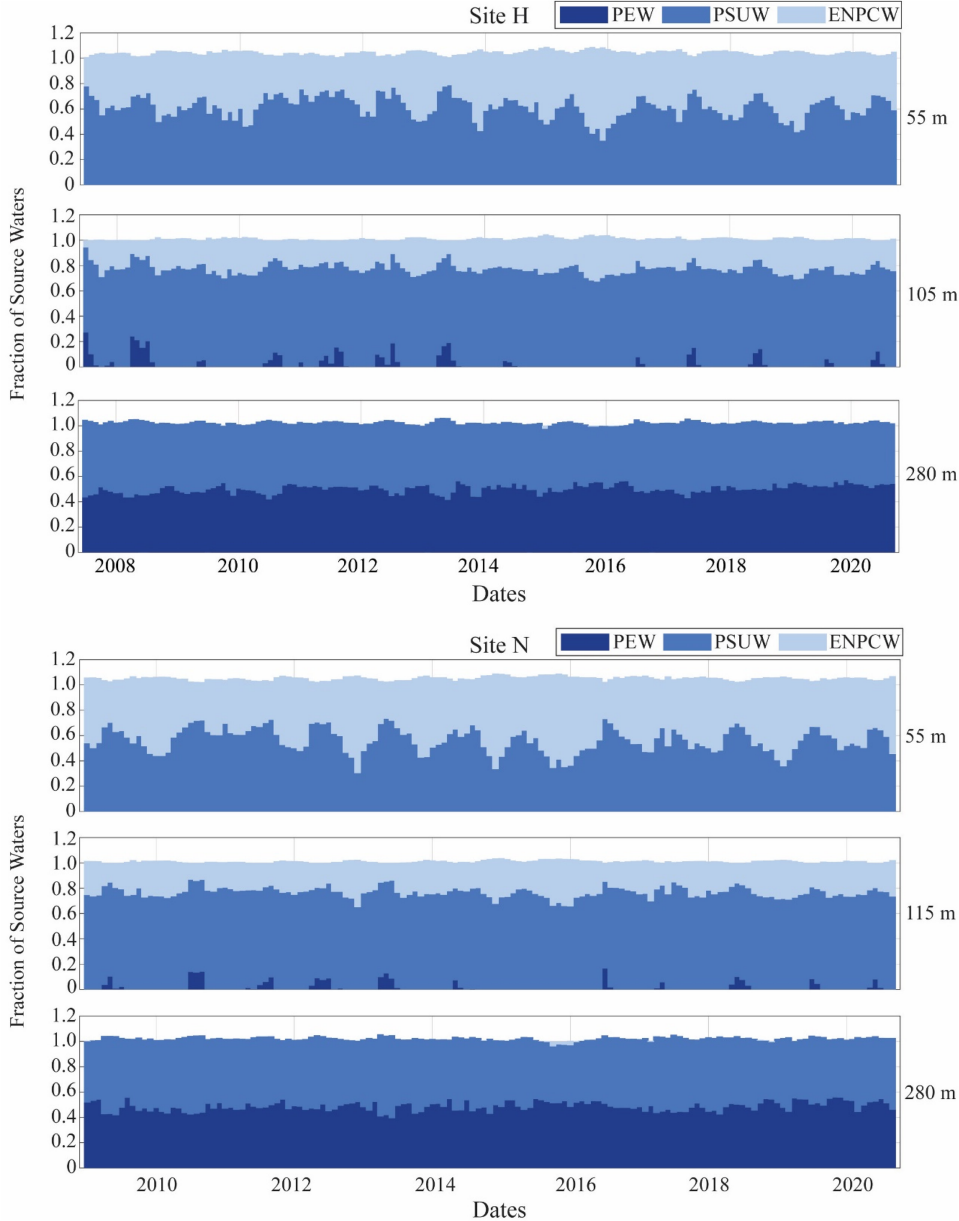
Fraction of ENPCW, PSUW, and PEW at selected depths. Fractions of the source waters present at the selected depths for each site (site H: 55 m, 105 m, and 280 m, site N: 55 m, 115 m, and 280 m).

The source water contributions were then quantified in two ways (Figure 3). First, the amounts of each source water present were quantified using the fractions given by the OMP analysis at the most representative depths (Figure 4, Table 1). For the PSUW and PEW, most representative depths were defined as the depths with the maximum average fraction (Table 1, Figure 3). The average fraction was calculated for each source water over all years at each depth and the maximum fraction across all depths was selected. For the ENPCW, the maximum average fraction was at 5 m, within a depth range prone to misclassification due to daily heating and cooling, therefore an alternate subsurface depth of 55 m was selected. This subsurface depth was selected to capture the strong ENPCW pulse at a depth where classification was more reliable. A second metric, referred to as the depth of each source water, was used to quantify the vertical changes in source water over time. A fraction for each source water was selected and the depth of that fraction was traced over time (Figure 3). Selected fractions at each site (and for each source water) were defined as the upper quartile fraction of the averaged fractions at each depth (Table 1, Figure 3).

**TABLE 1:** Selected depths and selected fractions of source waters. Selected depths (depth at which the maximum average fraction is found) and selected fractions (upper quartile fraction of the averaged fractions at each depth) for each of the source waters at sites H and N.

Source water	Selected depth		Selected fractions	
	Site H	Site N	Site H	Site N
PSUW	105 m	115 m	0.75	0.75
PEW	280 m	280 m	0.50	0.49
ENPCW	55 m	55 m	0.80	0.84

### 2.5 Seasonal and interannual modeling

Generalized additive models (GAMs) were constructed using the *mgcv* package (Wood, 2011) in R version 4.2.2 (R Core Team, 2022) to investigate whether and how seasonal and interannual variability in beaked whale presence could be explained by changes in the physical environment. The response variable, goose‐beaked whale presence, was quantified as the monthly total of click positive minutes. A Tweedie distribution was used to accommodate the zero‐inflated presence data. Nine explanatory variables were selected as potential influences on the seasonal and interannual variability in goose‐beaked whale presence: ENSO cycle (quantified by the ONI), a subsurface temperature value (temperature at 45 m), a subsurface salinity value (salinity at 45 m), the fraction of each water mass at the constant, most representative depths (Figure 4, Table 1), and the depths at which the upper quartile fraction for each water mass was traced (Figure 3, Table 1) were considered for the models.

Prior to running any of the models, highly collinear variables were removed. Correlation was tested using Pearson's correlation coefficient to get pairwise comparisons between individual environmental variables (Pearson's correlation coefficient >0.7) (Figure S1) (Dormann et al., 2012) and generalized variance inflation factors (GVIF) to measure each variable's multicollinearity (GVIF values <3 were achieved; Tables S3 and S4; Zuur et al., 2009). GVIF values were calculated using the *corvif* function (Zuur et al., 2009) in R version 4.2.2 (R Core Team, 2022). Highly collinear variables were individually removed based on their GVIF value and the pairwise variables they correlated with (Figure S1) until multicollinearity was no longer problematic. The variables that were not collinear were then fitted with the appropriate smoothing function. Fits were assessed by modeling goose‐beaked whale presence and individual environmental variables with different smooths or linear fits and comparing AIC values. For site H, the explanatory variables ENSO, the fraction of PSUW, and the depth of ENPCW remained, and were fitted linearly. The depth of PEW and fraction of PEW were estimated with a modified thin‐plate regression spline and the subsurface salinity values were estimated with a cubic spline. For site N, the explanatory variables ENSO, the depth of ENPCW, and the fraction of PEW, were estimated with a cubic‐spline modified thin‐plate regression spline. The depth of PEW, fraction of PSUW, and subsurface salinity were estimated with a modified thin plate regression spline. The smoothing parameters were set using the “Restricted Maximum Likelihood” (REML) method to optimize the level of smoothing (Marra & Wood, 2011). Backward and forward selected models were run to ensure all combinations of environmental variables and presence were assessed. Best model fits were assessed using the Akaike information criterion (AIC).

Goose‐beaked whale acoustic presence during La Niña, neutral, and El Niño events was further analyzed by running a Kruskal–Wallis test (Hollander & Wolfe, 1973), a non‐parametric test for a significant difference between mean groups, in R version 4.2.2 (R Core Team, 2022). ENSO events were further divided into weak, medium, and strong El Niño (weak: 0.5–1.5, medium: 1.5–2.5, strong: +2.5 temperature anomalies) and La Niña events (weak: −0.5 through −1.5, medium: −1.5 through −2.5 temperature anomalies). Post‐hoc testing was done using the Dunn test, part of the *rstatix* package (Kassambara, 2023) in R version 4.2.2 to identify which ENSO events had statistically significant differences in goose‐beaked whale acoustic presence. The Benjamini‐Hochberg method was applied to the Dunn tests to account for the numerous pairwise tests run and adjust the *p*‐values for type I error accordingly.

## 3 RESULTS

### 3.1 Beaked whale seasonality

**FIGURE 5:**
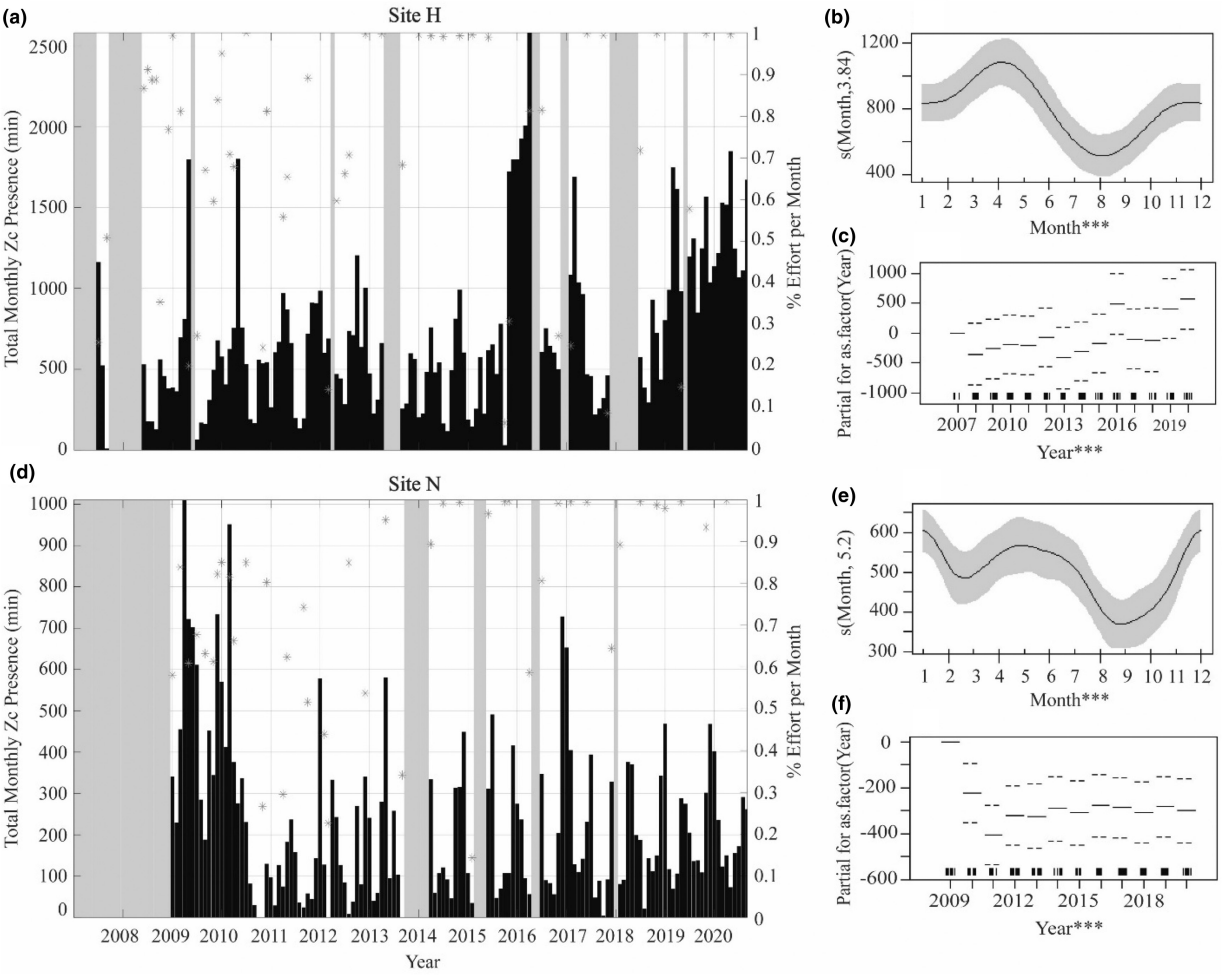
Total monthly goose‐beaked whale (Zc) presence. Top: Site H. Bottom: Site N. (a and d) Total monthly presence (minutes with acoustic presence recorded). Percent effort is on the right *y*‐axis and marked with gray stars over each month with less than full effort. Grayed out times on the plot show gaps in recording effort. GAM results documenting seasonality (b and e) and interannual variability (c and f) of goose‐beaked whale presence. Rug at the bottom of the plots denotes distribution. The categorical *p*‐value significance for each predictor variable is denoted in the figure by ***p < .001; **p < .01; and *p < .05. Model fit (black line) with confidence intervals (shaded area for smooth terms and dashed line for factors).

Seasonal trends in goose‐beaked whale presence were apparent in the acoustic recordings at both site H and N (Figure 5), with seasonal variability particularly strong at site H. At site H, acoustic presence was highest during the spring to early summer months, with a secondary high in presence during winter (Figure 5, Table S5). At site N, acoustic presence was elevated from the spring to early summer months and from late fall to winter (Figure 5, Table S5). At both sites, goose‐beaked whale presence dropped during mid‐summer and remained low through the early fall.

### 3.2 Seasonal variability of goose‐beaked whale acoustic presence and oceanography

**FIGURE 6:**
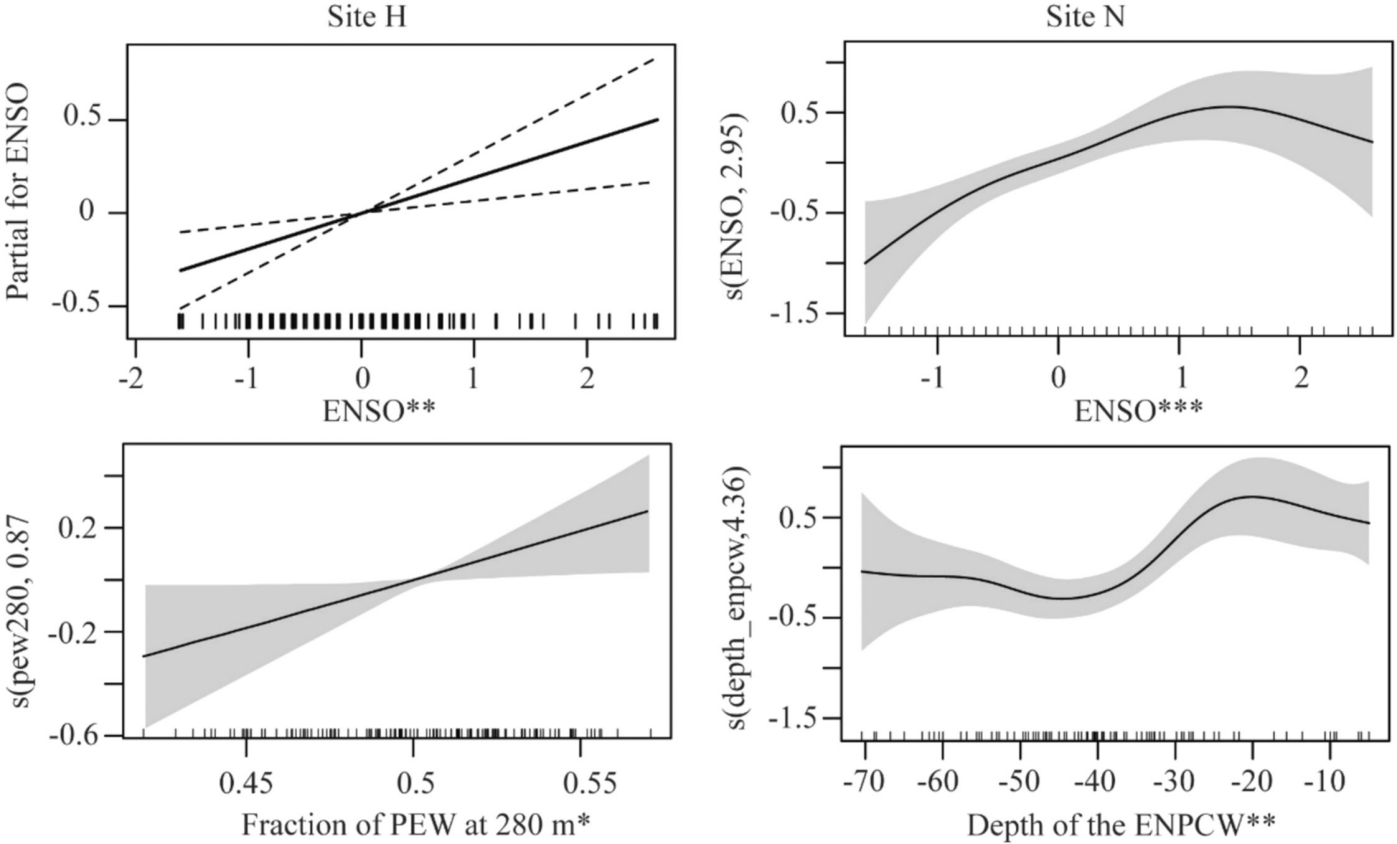
Seasonality and inter‐annual variability of goose‐beaked whale presence in relation to source waters and ENSO. Modeled relationships of the probability of goose‐beaked whale presence in relation to the significant environmental variables (splines as the black line with dashed or shaded area as the 95% confidence interval). Rug at the bottom of the plots denotes distribution. The categorical *p*‐value significance for each predictor variable is given as ***p < .001; **p < .01; and *p < .05.

GAMs were applied to monthly goose‐beaked whale presence data to investigate whether and how seasonal changes in the environment explained seasonal and interannual changes in their presence. The final habitat model for site H included the fraction of PEW (estimated with a modified thin plate regression spline) and the ENSO cycle (with a linear fit) as explanatory variables (Table S6, Figure S4). Goose‐beaked whale presence increased with stronger contributions of PEW in midwater depths (Figure 6). The final habitat model for site N included the depth of ENPCW and the ENSO cycle (both estimated with a cubic spline) (Table S6, Figure S4). As the depth of the ENPCW shoaled, goose‐beaked whale presence increased (Figure 6). Within the SCB, ENPCW followed a strong seasonal pattern (Figures S2 and S3, Table S7). Unlike PSUW and PEW that were present year‐round, ENPCW increased during the winter/spring (Bograd et al., 2019).

**FIGURE 7:**
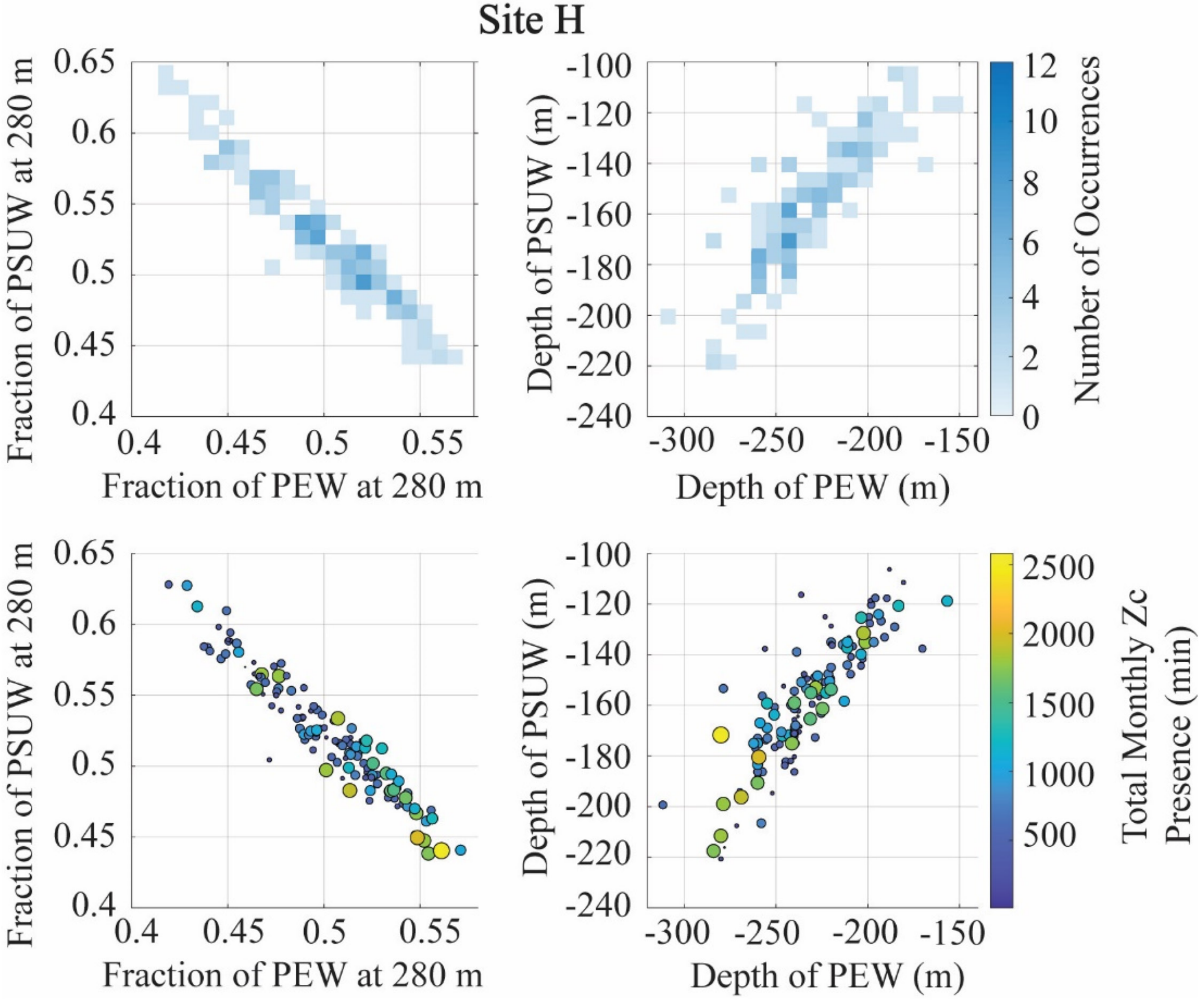
Interactions between the fraction and depth of PEW and PSUW at 280 m in relation to goose‐beaked whale (Zc) acoustic presence. Top: Fraction of PEW vs. the fraction of PSUW at 280 m and the depth of the PEW and the depth of the PSUW, with color indicating frequency of these events occurring. Bottom: Goose‐beaked whale presence during these varying conditions, with the amount of presence represented by the size and color of the dots. Larger, more yellow dots represent instances of higher goose‐beaked whale presence.

**FIGURE 8:**
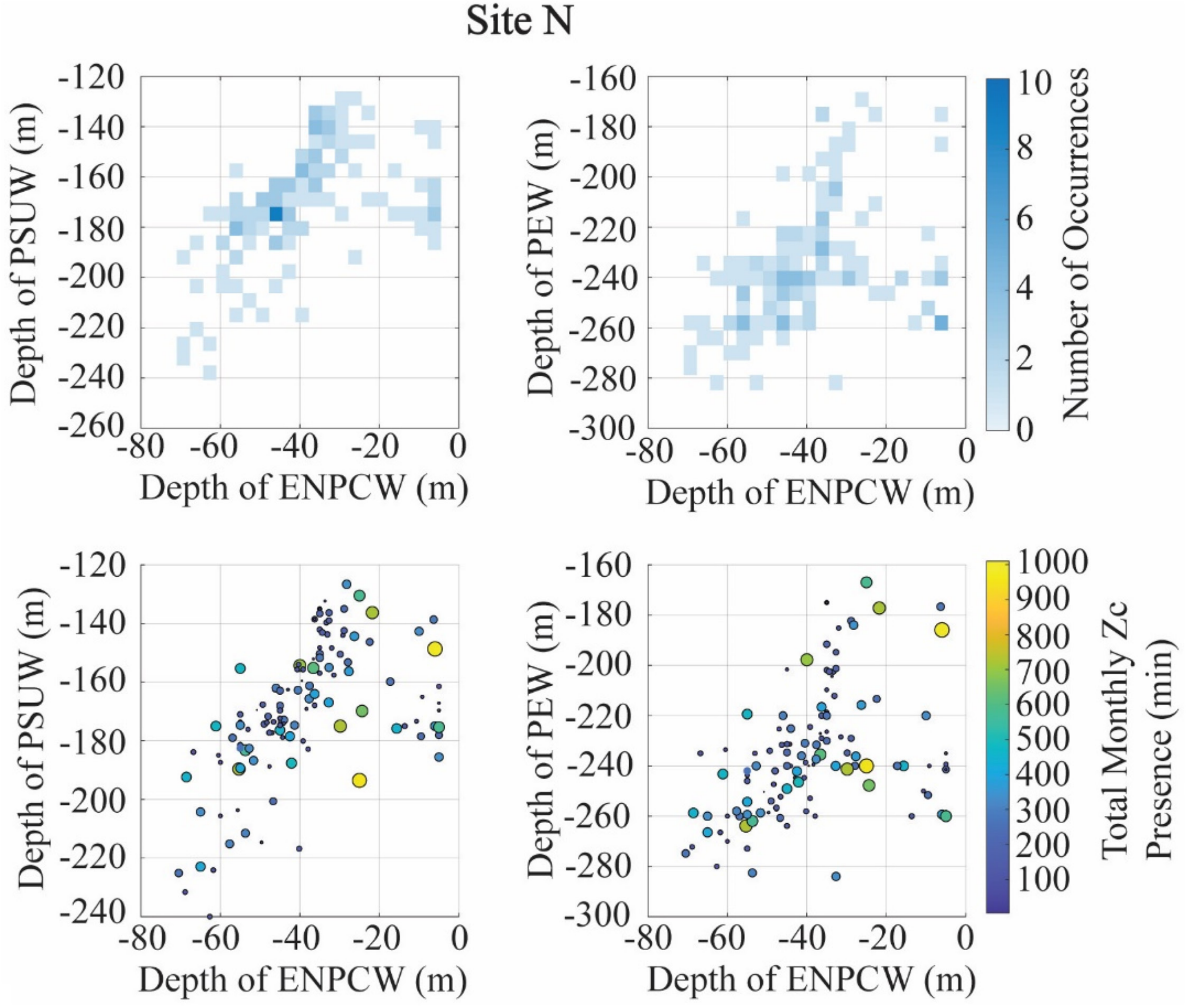
Interactions between the depth of the ENPCW and the other source waters in relation to goose‐beaked whale (Zc) acoustic presence. Top: Depth of the ENPCW in relation to the depth of the PSUW and PEW and the depth of the PSUW in relation to the depth of the PEW, with colors indicating the frequency of the depth pairs occurring. Bottom: Goose‐beaked whale presence during these varying depth pairs, with the amount of presence represented by the size and color of the dots. Larger, more yellow dots represent instances of higher goose‐beaked whale presence.

Of all the source water variables tested in our models, understanding the fraction of PEW at 280 m (at site H) and the depth of the ENPCW (at site N) in the context of the other source waters provides insights into the regional changes in the physical oceanography. At site H, when there was an increased fraction of PEW at 280 m, the fraction of PSUW at that depth decreased (Figure 7). It is during these times of high PEW and low PSUW that we noted high presence of goose‐beaked whales (Figure 7). At site N, we observed that when the depth of the ENPCW shoaled (disappearing from the region), the upper boundaries of the PSUW and PEW rose, filling the space left by the ENPCW (Figure 8). Increased presence of goose‐beaked whales was recorded when the ENPCW was shallow and thus in turn, the depths of the PSUW and PEW were also shallower (Figure 8).

### 3.3 Interannual variability of goose‐beaked whale acoustic presence and oceanography

**FIGURE 9:**
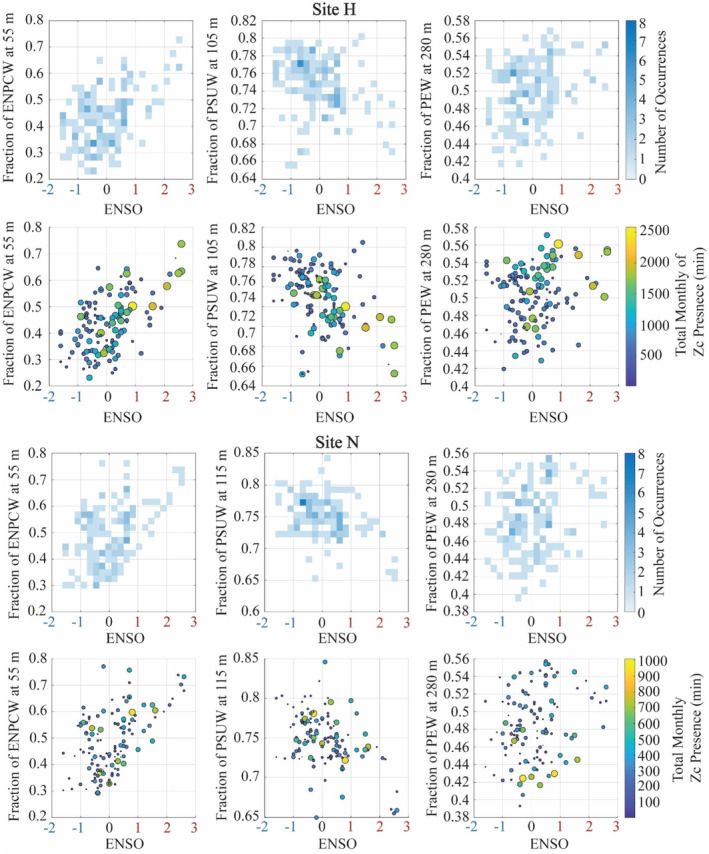
ENSO influence on the fraction of present source waters at selected depths and related goose‐beaked whale presence. For both sites H (top) and N (bottom)—top plots are colored by the number of occurrences when various fractions of each source water was present during El Niño/La Niña events. El Niño events are labeled red, neutral in black, and La Niña events in blue on the *x*‐axis. Darker blues indicate a more frequent occurrence of that fraction of source water during that ENSO event. Bottom plots show goose‐beaked whale (Zc) presence during these varying fractions of each source water and ENSO events, with the amount of presence represented by the size and color of the dots. Larger, more yellow dots represent instances of higher goose‐beaked whale presence.

There was interannual variability in the acoustic presence of goose‐beaked whales, with increased presence during 2015–2016 and 2019–2020 at site H and increased presence during 2009–2010 at site N (Figure 5). Across the timeseries, there was an overall increase in presence at site H over the years, with a sharp drop in presence in comparison to the 2009–2010 years at site N. The ENSO cycle was a significant predictor of this interannual variability at both sites H and N, with presence increasing during El Niño events and decreasing during La Niña (Figure 6, Table S6). The effects of ENSO events were also apparent in the source water depth and fraction at both sites. Year was a significant predictor of source water depth and fraction for all three source waters at both sites, except for the fraction of PSUW at site H (Figures S2 and S3, Table S7). During El Niño events, the fractions of ENPCW and PEW increased, while the fraction of PSUW decreased (Figure 9). When goose‐beaked whale presence was modeled, times of high presence correlated with El Niño events when there were higher fractions of ENPCW and PEW and less PSUW (Figure 9).

**FIGURE 10:**
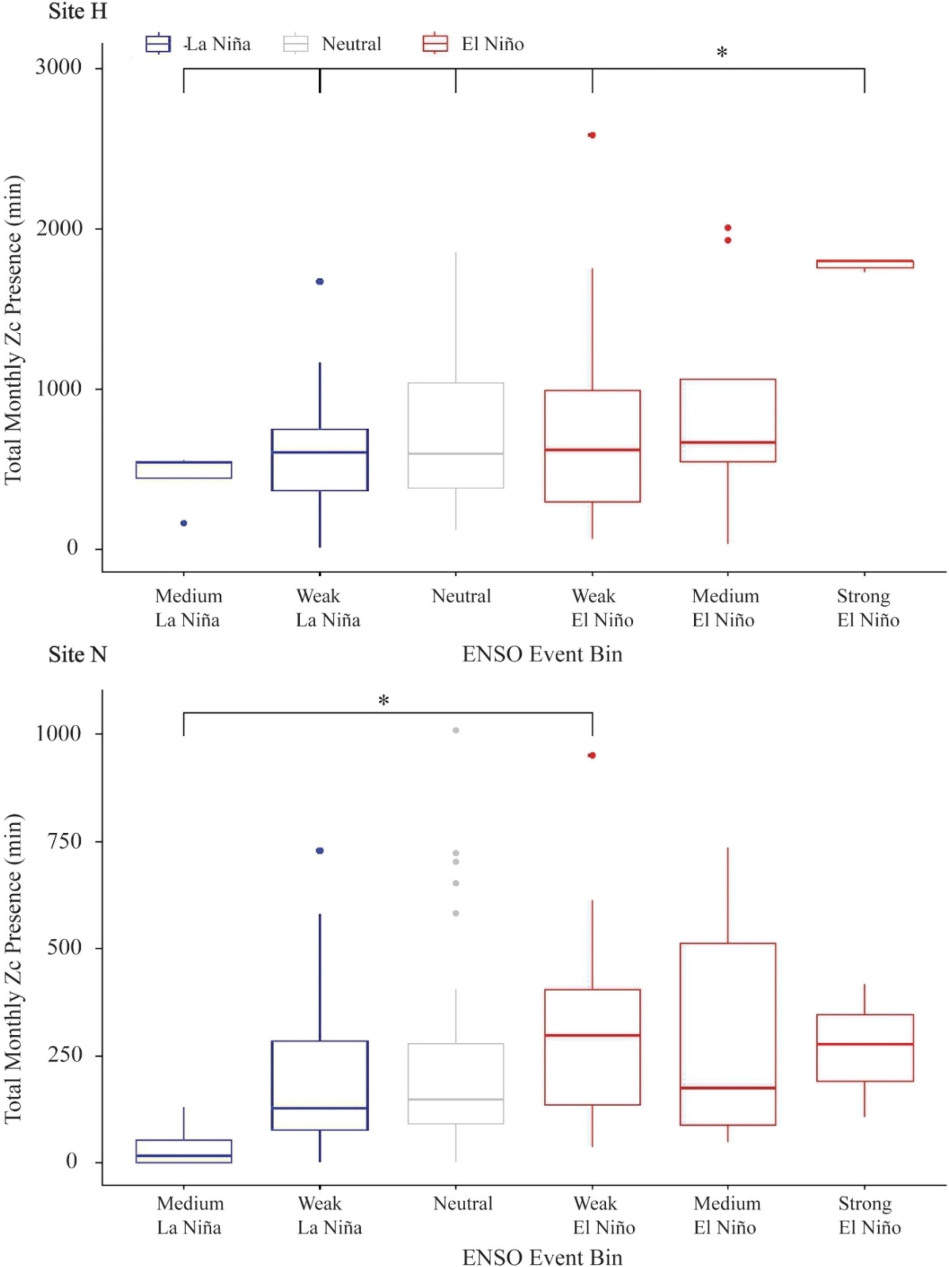
Goose‐beaked whale (Zc) acoustic presence during ENSO events. ENSO events are divided by color with La Niña events colored blue, neutral events gray, and El Niño events red. Significant pairs are indicated with a bracket with adjusted *p*‐value significance for each ENSO event pair denoted in the figure by  ***p < .001; **p < .01; and *p < .05.

Average goose‐beaked whale presence was significantly different between ENSO events and strengths (Kruskal–Wallis, *p* < .1 at site H, *p* < .05 at site N, Table S8). Post‐hoc testing revealed that this significant difference was driven by an increase in goose‐beaked whale presence during strong El Niño events at site H and weak El Niño events at site N (Figure 10, Table S9). At site H, goose‐beaked whale presence was significantly different between strong El Niño events and weak El Niño events, neutral times, and all La Niña events (Figure 10). At site N, goose‐beaked whale presence was significantly different during weak El Niño events and medium La Niña events (Figure 10).

## 4 DISCUSSION

A combination of multiple temporal scale fluctuations in the ocean's hydrography are correlated with seasonal and interannual variability in goose‐beaked whale acoustic presence. The seasonal increase in PEW at site H correlated with the springtime increase in goose‐beaked whale presence. At site N, the seasonal shoaling of the ENPCW depth correlated with the seasonal increase in goose‐beaked whale presence. It is important to note that while only one water mass variable per site was identified as significant, each source water should not be interpreted alone as they are highly correlated with each other. OMP analysis (Tomczak & Large, 1989) quantifies selected source waters as fractions of a whole. Therefore, an increase in one source water at a selected depth will be associated with the decrease of the other sources present at that same depth. This also applies to the changes in the vertical distribution of source waters: as the source waters at the surface deepen or expand to deeper depths, the source waters below must also change. On interannual time scales, El Niño and La Niña events influenced goose‐beaked whale presence, with increased presence recorded at both sites during El Niño events.

### 4.1 Seasonal variability in source waters and goose‐beaked whale acoustic presence

Increased fractions of PEW reflect a stronger influence of the warm, salty California Undercurrent (Lynn & Simpson, 1987). Along with an increase in the fraction of PEW, there is also a deepening of PEW that correlated with times of high goose‐beaked whale presence in the late winter/spring. While the depth of PEW was not significant in the seasonal model, high presence of goose‐beaked whales during an increased fraction and deeper depth of PEW could also indicate an increased volume of PEW and therefore an overall stronger influence of the California Undercurrent. The late winter/spring peak in goose‐beaked whale presence corresponded to times when there were increased fractions of both PSUW and PEW and a decreased fraction of ENPCW. These changes in source water are consistent with more nutrient‐rich waters being brought into the region, both at the surface and at depth (Lynn & Simpson, 1987). More nutrient‐rich waters could increase productivity in the region, potentially causing distributional shifts in the deep‐sea prey that goose‐beaked whales are following.

A shoaling or deepening in the ENPCW corresponded to its disappearance or reappearance at the surface, respectively. When present, the ENPCW brings warm, salty, nutrient‐poor, low‐oxygenated water at the surface (Bograd et al., 2019). In the absence of the ENPCW there is an increase in the fractions of and shallower depth of the PSUW and PEW. High goose‐beaked whale presence corresponded to times of the year when the ENPCW was fading or absent from the region. While the significantly correlated variables differed between sites, both sites indicated a goose‐beaked whale preference for nutrient‐rich water and decreased presence when nutrient‐poor waters dominated at the surface. While the influx of nutrient‐poor ENPCW is seen primarily at the surface and goose‐beaked whales are foraging near the seafloor, changes in the primary productivity near the surface may have bottom‐up impacts on prey availability. These shifts in hydrography are likely driving prey distribution and densities, especially given the spatially heterogeneous distribution of prey in the SCB (Benoit‐Bird et al., 2020; Southall et al., 2019), which could be the real driver of the goose‐beaked whales occurrence. Further work investigating the spatial and temporal variability in prey are necessary for assessing habitat quality and foraging patterns (Benoit‐Bird et al., 2020; Southall et al., 2019).

### 4.2 Sonar impacts on goose‐beaked whale acoustic presence

There are high numbers of goose‐beaked whale click‐positive minutes throughout the year at both sites, with an increase during the winter/spring and drop in presence during the summer. These findings are consistent with visual surveys that recorded fewer goose‐beaked whale sightings during the summer months (Curtis et al., 2020). Together, acoustic and visual data indicate that SOAR is an important foraging ground for goose‐beaked whales and provide further evidence of both resident and non‐resident groups inhabiting the region (Curtis et al., 2020; Schorr et al., 2014). While no resulting mass strandings associated with MFAS have been recorded in the SOAR region, tagging studies and visual surveys suggest exposure to MFAS results in altered foraging behaviors, decreased reproductive success, and an increased risk of decompression sickness (Curtis et al., 2020; D'Amico et al., 2009; Falcone et al., 2017; Schorr et al., 2014). This suggests that sonar impacts in SOAR are cumulative rather than acute (Curtis et al., 2020; Falcone et al., 2017; Schorr et al., 2014). Resident goose‐beaked whales may be familiar with the noisier soundscape, a potential explanation for the continued use of the region despite the frequent MFAS (Curtis et al., 2020; Falcone et al., 2009). This could put potential non‐resident goose‐beaked whales that are not acclimated to the regular disturbances at a higher risk for sub‐lethal consequences when exposed to MFAS (Curtis et al., 2020; Falcone et al., 2009).

### 4.3 Interannual variability

Interannually, goose‐beaked whale presence was higher during El Niño events than during neutral or La Niña events. During ENSO events, there were clear, interannual changes in source water composition and distribution. El Niño events typically result in an influx of warm, tropical waters, a deepening of the pycnocline, and weakened upwelling (Jacox et al., 2015). This decrease in the seasonal upwelling weakens or stops the input of deeper, nutrient‐rich waters from reaching the surface (Jacox et al., 2015). These changes in upwelling can be seen in the source water fractions and vertical distributions. During El Niño events, there is a strengthening in the California Undercurrent (Bograd et al., 2019), documented here as a higher fraction of PEW at 280 m. This increase in PEW is not seen at the surface, reflecting the weakened upwelling. In fact, surface waters are dominated by shallow source waters during El Niño events (Jacox et al., 2015). This is further demonstrated by the increase in the fraction of ENPCW at 55 m during El Niño events. During El Niño events, changes at the surface (stronger, deeper ENPCW influences) that alter vertical mixing may be relevant to these deep divers. Despite the increase in the nutrient‐poor ENPCW waters at the surface during El Niño events, the stronger contribution of nutrient‐rich PEW at mesopelagic depths likely constrains goose‐beaked whales and their prey to specific sites.

ENSO‐driven changes in the source water distributions and fractions can influence the entire food web, not just top predators, ultimately impacting the prey availability and distribution for goose‐beaked whales. El Niño driven changes in temperature and nutrient availability due to the weakened upwelling may force tropical species northward and offshore species inshore (Ohman et al., 2022). Therefore, despite drastic drops in primary productivity during El Niño events, it is possible that goose‐beaked whale prey species are moved into the SCB, aggregating at sites H and N as their habitat fluctuates. The strength of ENSO events, especially El Niño events, may also influence how prey is aggregated in these areas. Further research on goose‐beaked whale prey preference and prey spatio‐temporal distributions will be necessary to gain a more complete understanding of the bottom‐up factors that influence goose‐beaked whale prey and likely drive the predator's response.

### 4.4 Additional considerations and future work

There are some limitations to this study that must be noted. It is important to acknowledge that acoustic data can only record goose‐beaked whale presence when the animals are actively echolocating within the recording range of the HARP. Goose‐beaked whales only echolocate during their deep foraging dives (Baird, 2019). This caveat does not raise concerns for underrepresentation in goose‐beaked whale presence because goose‐beaked whales are likely consistently drawn to these sites to forage.

It must also be noted that OMP analysis is limited to the upper 500 m due to the limitations of the modeled environmental data and the end‐members that define the source waters (Bograd et al., 2019). While goose‐beaked whales can dive and forage much deeper than 500 m, previous studies support the utility of using surface, subsurface, and static environmental variables for describing goose‐beaked whale presence (Virgili et al., 2022). Correlations of goose‐beaked whale presence with water mass fluctuations shown by the OMP analysis are consistent with the hypothesis that changes in the surface waters impact animals even at depth. These environmental changes likely influence prey densities and distribution that then dictate goose‐beaked whale presence in the region.

Finally, this study addresses the oceanographic conditions that are suitable for goose‐beaked whale presence and highlights the value of continuing long‐term PAM, especially as a method for studying such an elusive species. Along with the addition of prey measurements, a consideration of human impacts could provide a more complete explanation for fluctuations in goose‐beaked whales in the SCB region. Goose‐beaked whales have clear behavioral responses to increased anthropogenic noise (Cox et al., 2006; D'Amico et al., 2009; Filadelfo et al., 2009). Goose‐beaked whales may leave the area and end foraging dives prematurely, suffer injury from ascending to the surface too quickly, or experience tissue damage when exposed to loud, anthropogenic sounds such as MFAS (Cox et al., 2006). With both sites within the US Navy's Southern California Range Complex, incorporating anthropogenic noise impacts into subsequent analyses could help further explain the variability in presence at these sites. It also demonstrates the importance of monitoring the seasonal and interannual patterns in goose‐beaked whale presence. Establishing when goose‐beaked whales are present and the conditions that attract them to the SCB are vital to implement successful mitigation efforts.

## AUTHOR CONTRIBUTIONS

**Clara M. Schoenbeck:** Conceptualization (equal); formal analysis (equal); methodology (equal); writing – original draft (equal). **Alba Solsona‐Berga:** Conceptualization (equal); data curation (equal); formal analysis (equal); methodology (equal); supervision (lead); writing – review and editing (equal). **Peter J. S. Franks:** Conceptualization (equal); methodology (equal); supervision (equal); writing – review and editing (equal). **Kaitlin E. Frasier:** Conceptualization (equal); supervision (equal); writing – review and editing (equal). **Jennifer S. Trickey:** Data curation (equal); writing – review and editing (equal). **Catalina Aguilar:** Data curation (equal); writing – review and editing (equal). **Isaac D. Schroeder:** Methodology (equal); writing – review and editing (equal). **Ana Širović:** Data curation (equal); funding acquisition (equal); writing – review and editing (equal). **Steven J. Bograd:** Methodology (equal); writing – review and editing (equal). **Ganesh Gopalakrishnan:** Methodology (equal); writing – review and editing (equal). **Simone Baumann‐Pickering:** Conceptualization (equal); data curation (equal); formal analysis (equal); funding acquisition (lead); methodology (equal); supervision (lead); writing – review and editing (equal).

## CONFLICT OF INTEREST STATEMENT

The authors have no conflicts of interest to declare.

## STATEMENT OF INCLUSION

Our study brings together a variety of collaborators that have extensive experience with the California Current Ecosystem. Together, collaborators worked to provide research in the interest of government and academic stakeholders. We would like to acknowledge that those invested in the California Current Ecosystem are not limited to government and academic stakeholders this research catered to, but also includes indigenous people, commercial users, and recreational use by the general public. It is our aim that our work contributes towards protected species management and conservation.

## Supporting information


Appendix S1.

## Data Availability

Acoustic data are available from the Dryad Digital Repository: https://datadryad.org/stash/share/24r5Ke2rXo9_g1Eo8f8KFwp_1hy__3O3KyJI0RqluN4.
