# Supplementary material for: Ziphius cavirostris presence relative to the vertical and temporal variability of oceanographic conditions in the Southern California Bight
Source: Ecol Evol. 2024 Jul 14;14(7):e11708. doi: 10.1002/ece3.11708 (PMC11246833; doi:10.1002/ece3.11708)
Supplement: Supplementary file 1 — Appendix S1. [file ECE3-14-e11708-s001.pdf]

SUPPLEMENTARY INFORMATION

Table S1: Summary of HARP deployment locations and depths.

Site	Location	Depth (m)
**H**	32° 50.76'N, 119° 10.57' W	1000
**N**	32° 22.21' N, 118° 33.85' W	1300

Table S2: Source water definitions and weights for the OMP analysis. Weights and definitions are modified from Bograd et al. (2019).

	Weight	ENPCW	PSUW	PEW
**Temperature (°C)**	10	18.77	6.88	9.47
**Salinity**	3	34.97	33.69	34.68
**Mass**	10	-	-	-

Table S3: Testing for collinearity between environmental variables for site H. GVIF values for all variables considered to be inputted into the habitat models, removing highly collinear variables (GVIF values < 3 were achieved). Bolded numbers indicate the variables that were not highly collinear in each round of testing.

**Site H**				
**Variable**	**GVIF**			
**Round #:**	**1**	**2**	**3**	**4**
PEW280	**1.19**	**1.18**	**1.18**	**1.17**
Zc_Pres	**1.29**	**1.24**	**1.24**	**1.22**
PSUW105	**1.62**	**1.57**	**1.57**	**1.56**
ENSO	**1.88**	**1.87**	**1.82**	**1.82**
S45	3.73	3.12	**2.14**	**2.07**
depth_pew	3.86	3.85	**2.83**	**2.39**

depth_enpcw	4.65	4.65	4.64	**2.04**
T45	21.30	7.75	6.00	
depth_psuw	13.46			
ENPCW55	26.08			

Table S4: Testing for collinearity between environmental variables for site N. GVIF values for all variables considered to be inputted into the habitat models, removing highly collinear variables (GVIF values < 3 were achieved). Bolded numbers indicate the variables that were not highly collinear in each round of testing.

**Site N**				
**Variable**	**GVIF**			
**Round #:**	**1**	**2**	**3**	**4**
PEW280	**1.26**	**1.22**	**1.21**	**1.17**
Zc_pres	**1.38**	**1.29**	**1.18**	**1.17**
S45	**1.83**	**1.75**	**1.59**	**1.32**
PSUW115	**1.90**	**1.77**	**1.72**	**1.53**
ENSO	**2.01**	**1.84**	**1.81**	**1.75**
depth_enpcw	**2.99**	**1.90**	**1.66**	**1.55**
depth_pew	3.27	3.20	**2.90**	**1.43**
depth_psuw	6.97	6.97	4.41	
ENPCW55	24.13	6.07		
T45	25.53			

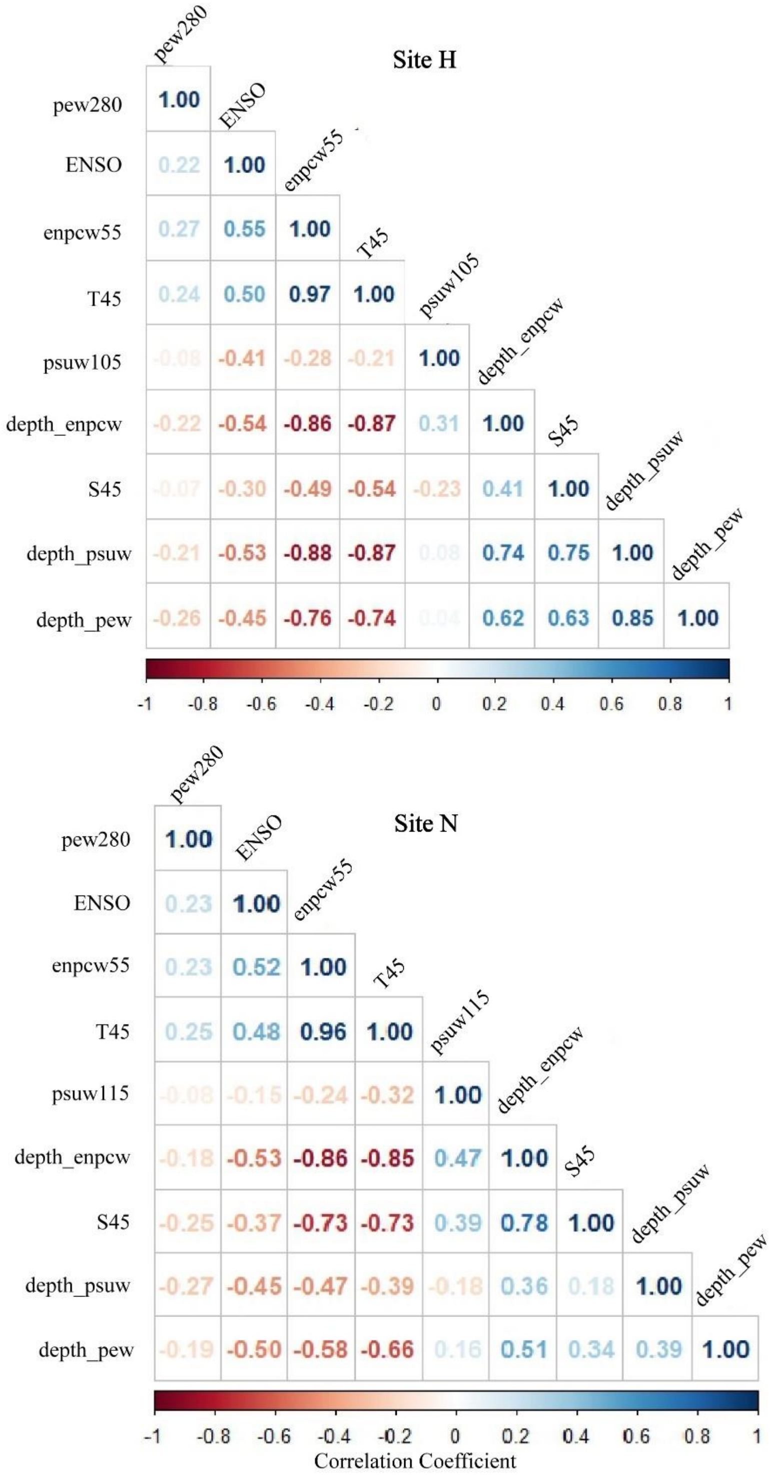

Figure S1: Correlation Plots for Sites H and N. Pairwise comparisons between individual environmental variables. High correlation coefficients (>0.7) were considered as highly correlated and considered alongside GVIF values when removing collinear variables and selecting variables to be inputted into the models.

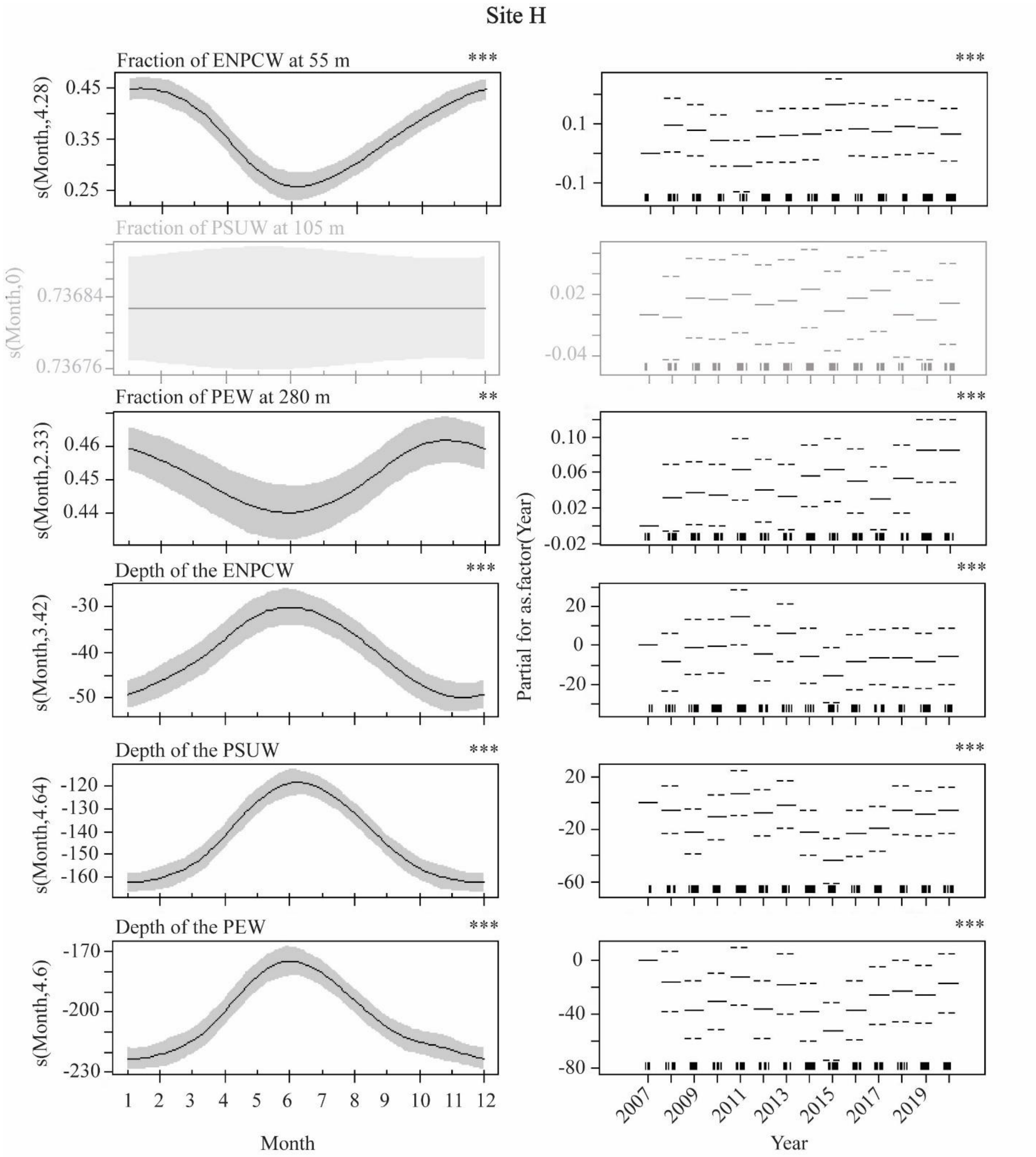

Figure S2: Seasonal and interannual trends in the fractions and depths of source waters at site H. Fraction and depth of each source water modeled by month (using a cubic-cyclic spline) and year (as a factor) to highlight seasonal and interannual trends in the fractions of source waters and vertical distributions. The categorical P-value significance for each predictor variable is given as *** $p < 0.001$; ** $p < 0.01$; * $p < 0.05$. Variables that came out as non-significant are grayed out.

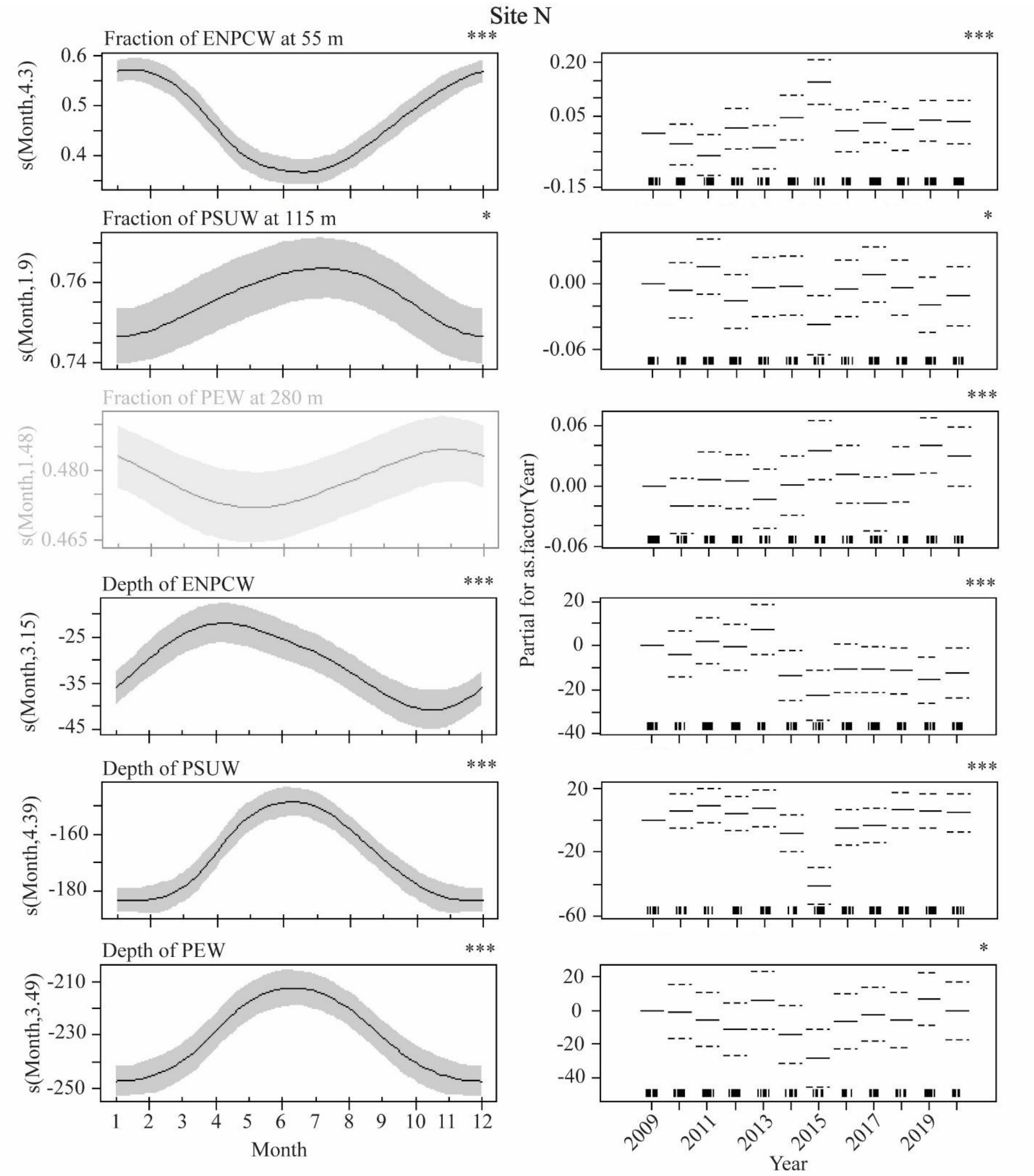

Figure S3: Seasonal and interannual trends in the fractions and depths of source waters at site N. Fraction and depth of each source water modeled by month (using a cubic-cyclic spline) and year (as a factor) to highlight seasonal and interannual trends in the fractions of source waters and vertical distributions. The categorical P-value significance for each predictor variable is given as *** $p < 0.001$; ** $p < 0.01$; * $p < 0.05$. Variables that came out as non-significant are grayed out.

Table S5: Summary of statistical outputs of the GAMs explaining monthly and yearly variability in goose-beaked whale presence. Goose-beaked whale presence was modeled with year (as a factor) and month (using a cubic-cyclic spline) as explanatory variables. The categorical P-value significance for each predictor variable is given as *** p < 0.001; ** p < 0.01; * p < 0.05.

Explanatory Variables	Site H		Site N	
**Temporal Factor**	**F**	**P-value**	**F**	**P-value**
**Month**	6.685	2.16e-09***	4.372	1.94e-05***
**Year**	3.73	5.14e-06***	4.124	8.49e-06***

Table S6: Summary of statistical outputs of the GAMs explaining monthly and interannual goose-beaked whale presence and multiple environmental conditions. Presence at site H is best explained by ENSO patterns and the fraction of PEW at 280 m. Presence at site N is best explained by ENSO patterns and the depth of ENPCW. The categorical P-value significance for each predictor variable is given as *** p < 0.001; ** p < 0.01; * p < 0.05.

**Site**	**H**							
**Explanatory Variable**	**Est**	**Error**	**t value**	**df**	**F**	**p-value**	**Dev. exp. (%)**	**R^2 adj**
ENSO	0.19176	0.06386	3.003	1	9.016	0.0032**	12.3	0.141
			**edf**	**Ref. df**	**F**	**p-value**		
PEW at 280 m			0.8663	9.0000	0.574	0.015*		
**Site**	**N**							
**Explanatory Variable**			**edf**	**Ref. df**	**F**	**p-value**	**Dev. exp. (%)**	**R^2 adj**
ENSO			2.951	3.697	5.530	0.00064***	23.8	0.217
ENPCW Depth			4.357	5.351	4.069	0.0016**		

Table S7: Summary of statistical outputs of the GAMs explaining monthly and yearly variability in the depths and fractions of source waters. Individual source water variables were modeled with year (as a factor) and month (using a cubic-cyclic spline) as explanatory variables. The categorical P-value significance for each predictor variable is given as *** p < 0.001; ** p < 0.01; * p < 0.05.

Environmental Variable	Fraction of ENPCW at 55 m			
Site	H		N	
Temporal Variable	F	P-value	F	P-value
Year	5.323	2.1e-07***	5.391	7.82e-07***
Month	16.95	<2e-16***	19.5	<2e-16***
Environmental Variable	Fraction of PSUW at 105 m		Fraction of PSUW at 115 m	
Year	1.02	0.437	2.034	0.031*
Month	<0.001	0.906	0.898	0.013*
Environmental Variable	Fraction of PEW at 280 m			
Year	5.439	1.32e-07***	3.818	0.000108***
Month	1.517	0.0017**	0.45	0.0725
Environmental Variable	Depth of ENPCW			
Year	5.021	5.84e-07***	4.499	1.25e-05***
Month	7.044	<2e-16***	4.125	7.19e-07***
Environmental Variable	Depth of PSUW			
Year	10.73	1.39e-14***	9.746	3.38e-12***
Month	22.92	<2e-16***	16.06	<2e-16***
Environmental Variable	Depth of PEW			
Year	5.736	5.18e-08***	2.311	0.0135*
Month	16.41	<2e-16***	7.518	<2e-16***

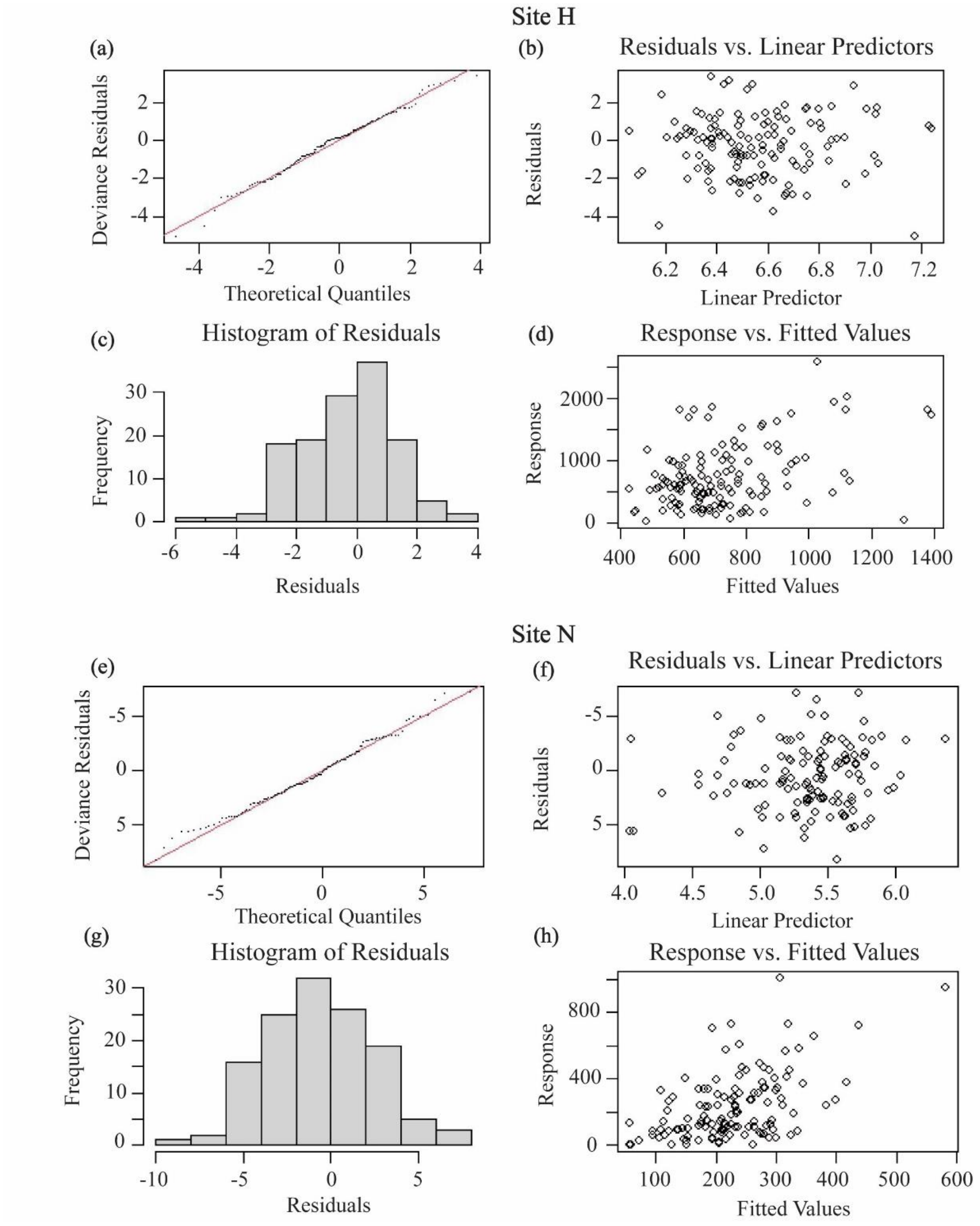

Figure S4: Gam.check plots for the habitat models for sites H and N. (a & e) Q-Q plots, (b & f) plot of residual values, (c & g) histogram of residuals, and (d & h) response vs. fitted values. These plots assess the fit of the GAMs explaining monthly and interannual variability in goose-beaked whale presence with multiple environmental variables.

Table S8. Summary of statistical outputs of the Kruskal-Wallis test explaining goose-beaked whale presence and ENSO events. The categorical P-value significance for each predictor variable is given as *** $p < 0.001$; ** $p < 0.01$; * $p < 0.05$; • $p<0.1$.

**Site**	**Chi-squared value**	**df**	**p-value**
H	9.9202	5	0.078 •
N	14.516	5	0.013*

Table S9. Summary of statistical outputs of the post-hoc Dunn test identifying the pairs of ENSO events when goose-beaked whale presence is significantly different. The Benjamini-Hochberg method was applied to account for type I error when running so many tests. The categorical P-value significance for each predictor variable is given as *** $p < 0.001$; ** $p < 0.01$; * $p < 0.05$.

		**Site H**			**Site N**		
**Pair of ENSO Events**		**Z statistic**	**p-value**	**Adjusted p-value**	**Z statistic**	**p-value**	**Adjusted p-value**
Med. La Niña	Weak La Niña	0.77	0.444	0.563	1.94	0.053	0.113
Med. La Niña	Neutral	1.13	0.257	0.479	2.35	0.019	0.076
Med. La Niña	Weak El Niño	1.11	0.266	0.479	3.18	0.0015**	0.022*
Med. La Niña	Med. El Niño	1.34	0.180	0.451	2.32	0.020*	0.076
Med. La Niña	Strong El Niño	2.76	0.0057**	0.043*	2.20	0.028*	0.083
Med. La Niña	Neutral	0.85	0.398	0.563	0.88	0.381	0.571
Weak La Niña	Weak El Niño	0.76	0.450	0.563	2.65	0.008**	0.060
Weak La Niña	Med. El Niño	1.06	0.287	0.479	1.01	0.313	0.521
Weak La Niña	Strong El Niño	2.84	0.0046*	0.043*	1.09	0.276	0.517
Neutral	Weak El Niño	0.02	0.986	0.986	2.06	0.039	0.098
Neutral	Med. El Niño	0.61	0.539	0.605	0.54	0.592	0.683
Neutral	Strong El Niño	2.57	0.0103*	0.046*	0.78	0.435	0.594

Weak El Niño	Med. El Niño	0.58	0.565	0.605	-0.70	0.485	0.606
Weak El Niño	Strong El Niño	2.51	0.012*	0.046*	-0.03	0.973	0.973
Med. El Niño	Strong El Niño	1.91	0.057	0.170	0.38	0.701	0.751

72
